# Site-directed mutagenesis identified the key active site residues of alcohol acyltransferase PpAAT1 responsible for aroma biosynthesis in peach fruits

**DOI:** 10.1038/s41438-021-00461-x

**Published:** 2021-02-01

**Authors:** Zhi-Zhong Song, Bin Peng, Zi-Xia Gu, Mei-Ling Tang, Bei Li, Mei-Xia Liang, Li-Min Wang, Xiao-Tong Guo, Jian-Ping Wang, Yu-Fen Sha, Hong-Xia Zhang

**Affiliations:** 1 The Engineering Research Institute of Agriculture and Forestry, Ludong University, 186 Hongqizhong Road, Yantai, 264025 China; 2 Key Laboratory of Molecular Module-Based Breeding of High Yield and Abiotic Resistant Plants in Universities of Shandong (Ludong University), 186 Hongqizhong Road, Yantai, 264025 China; 3 Institute of Botany, Jiangsu Province and Chinese Academy of Sciences, 1 Qianhuhoucun, Nanjing, 210014 China; 4 Yantai Academy of Agricultural Science, 26 Gangcheng West Street, Yantai, 265500 China

**Keywords:** Molecular engineering in plants, Complement, Plant physiology

## Abstract

The aroma of peach fruit is predominantly determined by the accumulation of γ-decalactone and ester compounds. A previous study showed that the biosynthesis of these aroma compounds in peach fruit is catalyzed by PpAAT1, an alcohol acyltransferase. In this work, we investigated the key active site residues responsible for γ-decalactone and ester biosynthesis. A total of 14 candidate amino acid residues possibly involved in internal esterification and 9 candidate amino acid residues possibly involved in esterification of PpAAT1 were assessed via site-directed mutagenesis. Analyses of the in vitro enzyme activities of PpAAT1 and its site-directed mutant proteins (PpAAT1-SMs) with different amino acid residue mutations as well as the contents of γ-decalactone in transgenic tobacco leaves and peach fruits transiently expressing PpAAT1 and PpAAT1-SMs revealed that site-directed mutation of H165 in the conserved HxxxD motif led to lost enzymatic activity of PpAAT1 in both internal esterification and its reactions, whereas mutation of the key amino acid residue D376 led to the total loss of γ-decalactone biosynthesis activity of PpAAT1. Mutations of 9 and 7 other amino acid residues also dramatically affected the enzymatic activity of PpAAT1 in the internal esterification and esterification reactions, respectively. Our findings provide a biochemical foundation for the mechanical biosynthesis of γ-decalactone and ester compounds catalyzed by PpAAT1 in peach fruits, which could be used to guide the molecular breeding of new peach species with more favorable aromas for consumers.

## Introduction

As an olfactory component of flavors, fruit aroma is an important index for fruit quality evaluation^1–4^. The unique aroma of different fruits depends on their volatile profiles, which are mainly composed of esters, lactones, aldehydes, and alcohol terpenoids^5,6^. In mountain papaya (*Vasconcellea pubescens* (Lenné et C. Koch) Badillo), the volatile compounds are mainly esters, with a low proportion of alcohols^7^. Whereas in apple (*Malaus domestica* Borkh.), the major volatile components are esters^8^. In some fruits, even a single compound, such as the key volatile 4-hydroxy-2,5-dimethyl-3(*2H*)-furanone in strawberry (*Fragaria* spp.), can constitute a characteristic aroma^9,10^. In peach (*Prunus persica* L.), γ-decalactone, catalyzed by PpAAT1, is the most important volatile compound and is responsible for the characteristic aroma of the fruits^11–14^.

PpAAT1 belongs to the plant BAHD superfamily of acyltransferases named after the four enzymes first discovered in the BEAT (benzoyl alcohol *O*-acetyltransferase), AHCT (anthocyanin *O*-hydroxycinnamoyl transferase), HCBT (anthranilate *N*-hydroxycinnamoyl/benzoyl transferase), and DAT (deacetyl vindoline 4-*O*-acetyltransferase) families^15^. In higher plants, the BAHD acyltransferase family catalyzes the biosynthesis of a huge diversity of natural products, including esters and amides, and can be identified by sequence homology and the universally conserved HxxxD and D(N)F(V)GWG motifs^16–18^. BAHD acyltransferase family members act on different substrates, and their HxxxD motif plays a pivotal role in the catalytic process. Site-directed mutation of histidine and aspartate residues with alanine in the HxxxD motif of vinorine synthase, a member of the BAHD acyltransferase family catalyzing acetyl–CoA- or CoA-dependent vinorine formation, led to the loss of its enzymatic activity^19^. Similar results were also observed with the substitution of H166 and D170 in the HxxxD motif of alcohol acyltransferase (VpAAT1), which catalyzes ester biosynthesis in mountain papaya^7,20^. However, the functions of other amino acid residues in the internal esterification and esterification of alcohol acyltransferases are largely known. PpAAT1, like other known AATs that also contain an HxxxD-type acyltransferase-like motif (HAMCD), could catalyze both the internal esterification reaction at the hydroxyl (–OH) and –CoA groups of 4-hydroxydecanoyl–CoA and the esterification reaction between alcohols and acyl-CoAs^14^.

In this study, the key amino acid residues responsible for both internal esterification and esterification of PpAAT1 were examined via site-directed amino acid residue mutation. We demonstrate that the amino acid residue H165 in the HxxxD motif and 18 other amino acid residues of PpAAT1 play a crucial role in maintaining its internal esterification and esterification activities, as verified via in vitro enzymatic activity and in vivo aroma biosynthesis analyses.

## Results

### Computer modeling and molecular docking analysis of PpAAT1

**Fig. 1:**
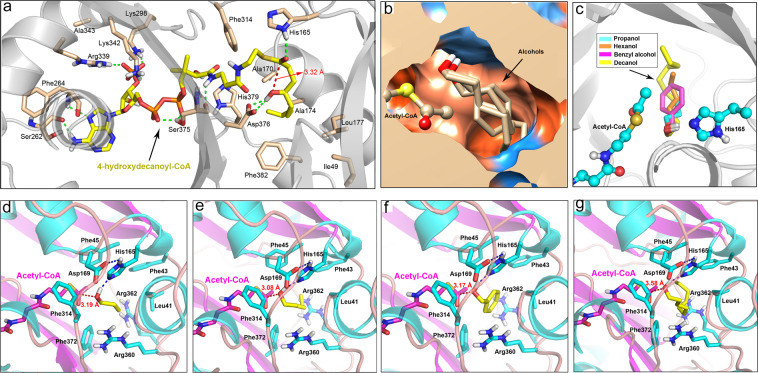
Computer modeling and molecular docking-based structure of PpAAT1. **a** Predicted key amino acid residues of PpAAT1 interacting with 4-hydroxydecanoyl–CoA during the internal esterification reaction. A total of 16 amino acid residues were identified. **b** Stereoview of the substrate binding pocket. Acetyl–CoA and the four alcohols (propanol, hexanol, benzyl alcohol, and decanol) were docked to the active center during the esterification reaction. **c** Stereoview of the active site. Alcohols were positioned between acetyl–CoA and H165 in the most reasonable binding conformation during the esterification reaction. **d** Orientation of the key amino acid residues catalyzing the esterification reaction between acetyl–CoA and propanol. **e** Orientation of the key amino acid residues catalyzing the esterification reaction between acetyl–CoA and hexanol. **f** Orientation of the key amino acid residues catalyzing the esterification reaction between acetyl–CoA and benzyl alcohol. **g** Orientation of the key amino acid residues catalyzing the esterification reaction between acetyl–CoA and decanol

To clarify the exact biochemical function of PpAAT1, we first performed computer modeling and molecular docking analyses. Stereochemical quality analysis showed that 99.2% of the amino acid residues of the PpAAT1 model structure were in a reasonable range. Verification by 3D protein modeling analysis showed that 86.8% of the amino acid residues scored above 0.2, which met the requirements of the evaluation procedure. Therefore, the final structure of the PpAAT1 protein was accepted for subsequent analyses. A total of 16 amino acid residues of PpAAT1 were predicted to be involved in the biosynthesis of γ-decalactone (Fig. 1a). To identify the amino acids participating in enzyme–substrate recognition in the esterification reaction, acetyl–CoA and the four alcohols were docked to the active center of PpAAT1 to form a binding conformation (Fig. 1b). Acetyl–CoA and four different alcohols (propanol, hexanol, benzyl alcohol, and decanol), which could potentially be used as substrates for the production of ester and were found in the high-aroma peach cultivar ‘Fenghuayulu’ in our previous study, were assessed^14^. In the PpAAT1–alcohol–acetyl–CoA complexes, the hydroxyl group was located between acetyl–CoA and amino acid residue His165 (Fig. 1c). To determine the amino acid residues possibly responsible for enzyme–substrate recognition in the esterification reaction, the binding mode of acetyl–CoA with propanol, hexanol, benzyl alcohol, and decanol was analyzed. A total of 9 candidate amino acid residues (Leu41, Phe43, Phe45, His165, Asp169, Phe314, Arg360, Arg362, and Phe372) possibly involved in the esterification reaction between acetyl–CoA and alcohols were identified (Fig. 1d–g). It is worth mentioning that residues His165 and Phe314 participate in both internal esterification and its reactions.

**Fig. 2:**
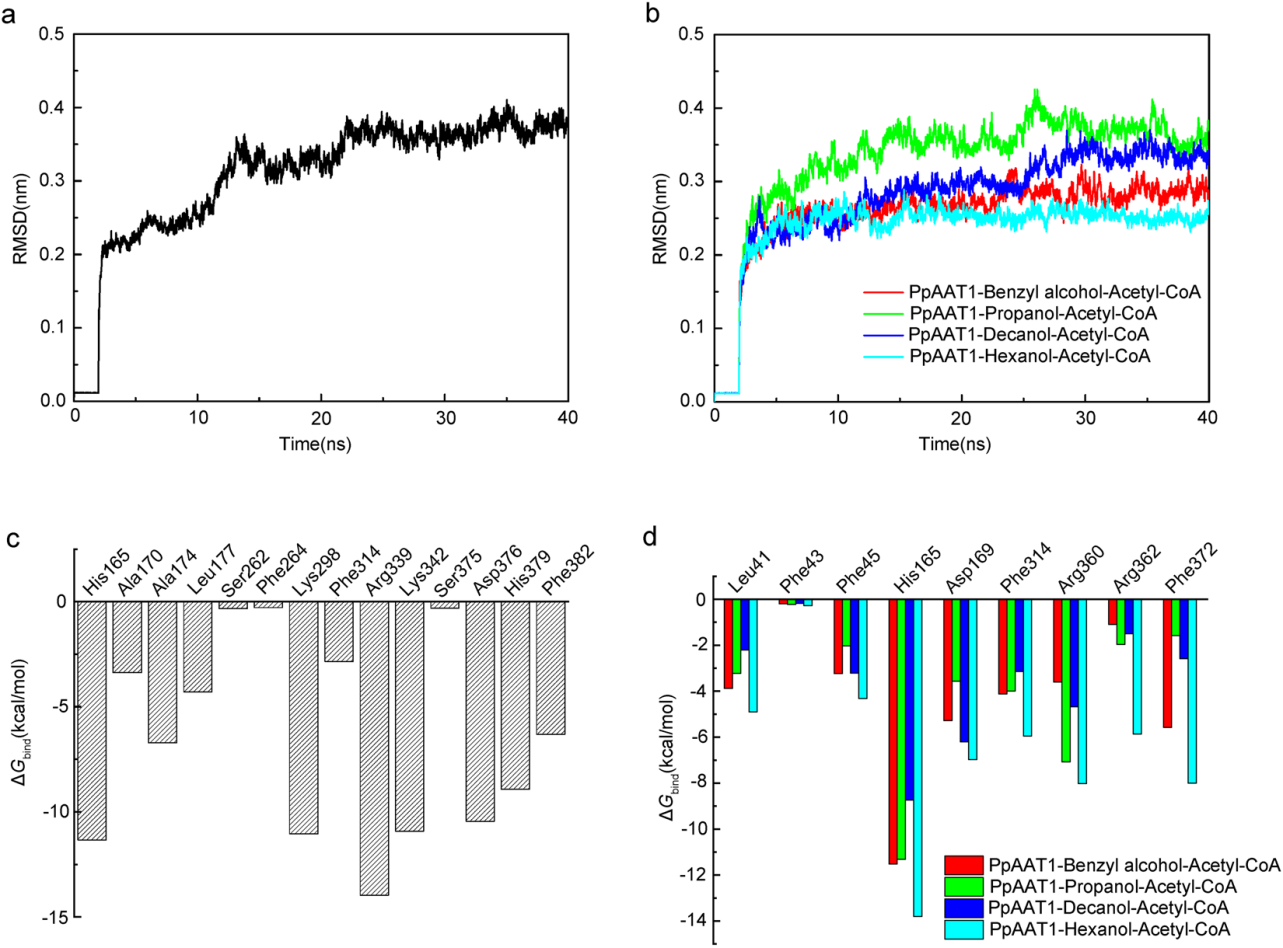
RMSD values of PpAAT1–ligand complexes and contribution of amino acid residues to Δ*G*_bind_ in the PpAAT1–alcohol–acetyl–CoA complexes. **a** RMSD values of the PpAAT1–4-hydroxydecanoyl–CoA complexes. **b** RMSD values of the four PpAAT1–alcohol–acetyl–CoA complexes. **c** Contribution of amino acid residues to Δ*G*_bind_ in the PpAAT1–4-hydroxydecanoyl–CoA complexes. **d** Contribution of amino acid residues to Δ*G*_bind_ in the PpAAT1–alcohol–acetyl–CoA complexes

We then evaluated the root mean square deviation (RMSD) values for the backbone atoms in their initial configuration as a function of simulation time for each investigated reaction system. No significant changes in the dynamics of PpAAT1–4-hydroxydecanoyl–CoA and four PpAAT1–alcohol–acetyl–CoA complexes were observed after 30 ns (Fig. 2a, b). The average RMSD values of the internal esterification and its reaction systems were 0.285 ± 0.011, 0.365 ± 0.014, 0.337 ± 0.011, 0.252 ± 0.007, and 0.372 ± 0.013 nm, respectively, suggesting that the stability of PpAAT1–ligand complexes was reached during the molecular dynamics simulations (MDS).

To estimate the relative binding free energy (Δ*G*_bind_) of the PpAAT1–ligand complexes, MM-GBSA (molecular mechanics–generalized Born surface area (MM–GBSA)) was performed. The van der Waals (vdW), solvation (polar and nonpolar), and electrostatic contributions were calculated to obtain detailed molecular information on the internal esterification and its reaction systems investigated (Table 1). We found that the vdW $\left( {\Delta H_{{\mathrm{MM}}}^{{\mathrm{vdW}}}} \right)$ value made the greatest contribution to the total free energy in each complex, and the interaction energy of the PpAAT1–hexanol–acetyl–CoA complex in the esterification reaction system was stable (−105.77 ± 3.75 kcal/mol). To further understand the free energy of the key amino acid residues that affect substrate binding in PpAAT1, Δ*G*_bind_ was decomposed using MM-GBSA. The results showed that the amino acid residues His165, Lys298, Arg339, Lys342, Asp376, and His379 contributed more than Ser262, Phe264 and Ser375, and His165 contributed more than Phe43 to substrate binding in the internal esterification and its reactions (Fig. 2c, d).

**Table 1:** MM-GBSA analysis of PpAAT1–ligand complexes (kcal/mol)

Complexes	$\Delta H_{{\mathrm{MM}}}^{{\mathrm{vdW}}}$	$\Delta H_{{\mathrm{MM}}}^{{\mathrm{ele}}}$	Δ*G*_sol-pol_	Δ*G*_sol-npol_	Δ*G*_bind_
PpAAT1–propanol-acetyl–CoA	−45.00 ± 1.38	−40.73 ± 6.19	20.73 ± 3.38	−12.20 ± 0.02	−77.51 ± 1.82
PpAAT1–benzyl alcohol–acetyl–CoA	−51.25 ± 2.34	−47.05 ± 3.95	23.74 ± 3.70	−13.71 ± 0.03	−88.96 ± 2.91
PpAAT1–decanol-acetyl–CoA	−40.47 ± 1.98	−35.66 ± 5.21	19.14 ± 2.93	−13.05 ± 0.07	−70.32 ± 2.25
PpAAT1–hexanol-acetyl–CoA	−60.08 ± 2.96	−47.04 ± 8.85	19.39 ± 1.43	−17.58 ± 0.04	−105.77 ± 3.75
PpAAT1–4-hydroxydecanoyl–CoA	−86.63 ± 4.22	−80.33 ± 11.09	23.83 ± 8.02	−13.10 ± 0.06	−156.96 ± 5.94

$\Delta H_{{\mathrm{MM}}}^{{\mathrm{vdW}}}$ corresponds to the van der Waals contribution, $\Delta H_{{\mathrm{MM}}}^{{\mathrm{ele}}}$ corresponds to the electrostatic contribution, Δ*G*_sol-pol_ corresponds to the polar solvation component, Δ*G*_sol-npol_ corresponds to the nonpolar solvation component, and Δ*G*_bind_ corresponds to the binding free energy of the protein–ligand complex

### Substitution of key amino acid residues decreases the enzymatic activity of PpAAT1 in vitro

To assess the individual contribution of each candidate amino acid residue to the internal esterification and its reactions of PpAAT1, computer modeling and molecular docking studies were further performed, and the function of each candidate amino acid residue possibly involved in the internal esterification and its reactions of PpAAT1 was examined via site-directed mutagenesis. Our previous study demonstrated that substitutions of Ile49 and Ala343 in PpAAT1 led to decreased γ-decalactone production in a low-aroma peach cultivar^14^. Therefore, we substituted the remaining 14 candidate amino acid residues possibly involved in the internal esterification reaction and the 9 candidate amino acid residues possibly involved in the esterification reaction in the present study.

**Fig. 3:**
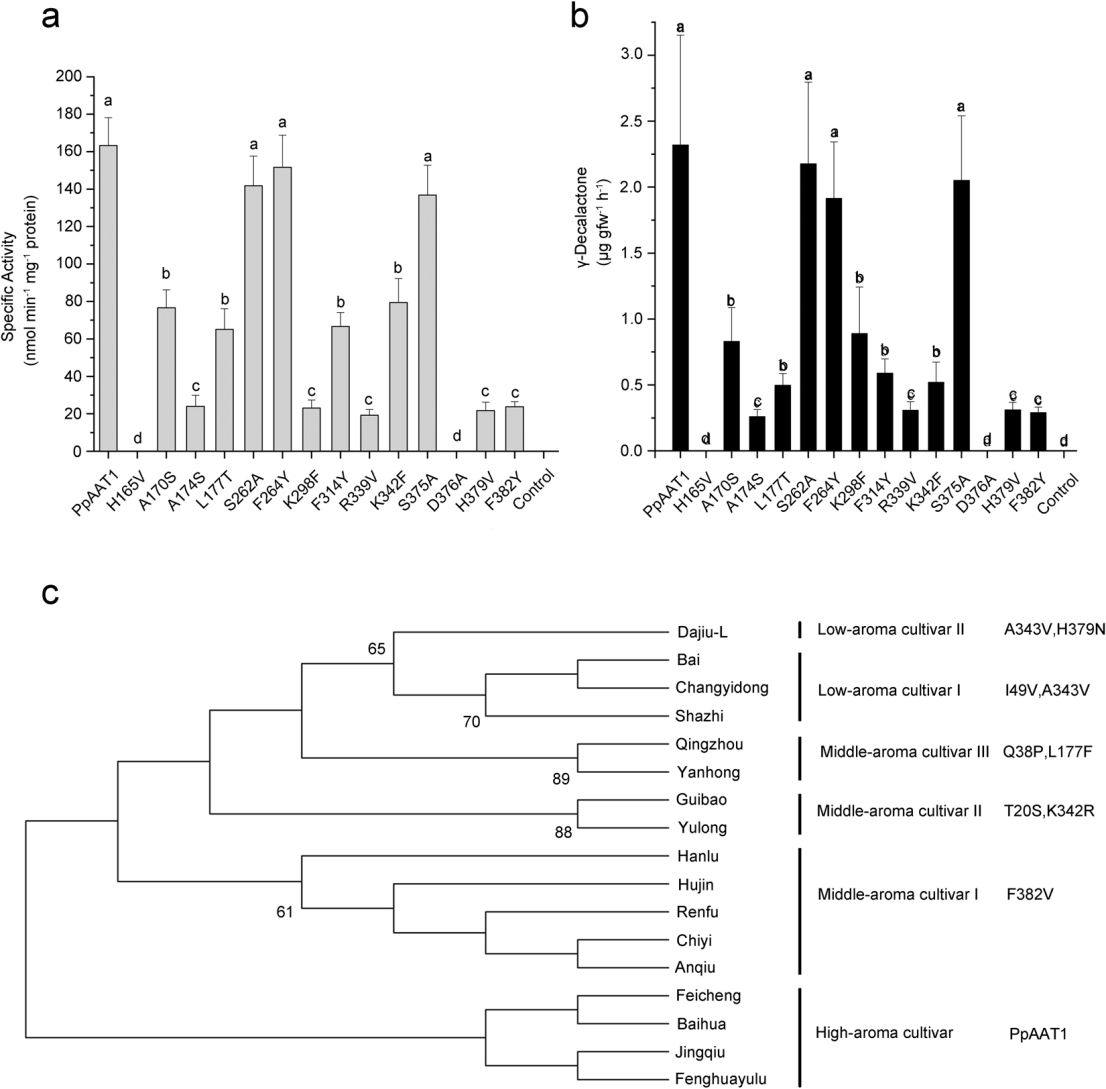
Enzymatic activity, γ-decalactone production, and phylogenetic analyses. **a** In vitro activities of PpAAT1 and PpAAT1-SMs in the production of γ-decalactone. **b** γ-Decalactone contents detected in the leaves of transgenic *N. benthamiana* transiently expressing *PpAAT1* and *PpAAT1*-*SM*s. Data are presented as the means ± SE (*n* = 3). Letters represent significant differences at *P* ≤ 0.05 as determined using ANOVA followed by Fisher’s LSD test. **c** Phylogenetic analysis of the amino acid sequences of PpAAT1 from different peach cultivars. The numbers on the nodes are support values. The descriptions on the right are the amino acid residues substituted in the corresponding varieties

PpAAT1 and its site-directed mutant proteins (PpAAT1-SMs) were heterologously expressed in *Escherichia coli*, and the enzymatic activities of the purified PpAAT1 and PpAAT1-SMs in the internal esterification reaction with 4-hydroxydecanoyl–CoA as the substrate were first investigated in vitro. Among the 14 candidate amino acid residues, substitutions of S262, F264, and S375 did not cause any significant effects on the enzymatic activities of the corresponding PpAAT1-SMs. However, substitutions of all 11 candidate amino acid residues possibly involved in the internal esterification reaction dramatically decreased the enzymatic activities of the corresponding PpAAT1-SMs, especially the substitutions of H165 and D376, which led to the total loss of enzymatic activities (Fig. 3a). Consistent results were also observed with the enzyme kinetic analyses. Substitutions of S262, F264, and S375 did not cause any significant effects on the affinity (*K*_m_ values) and enzyme efficiency (*K*_cat_/*K*_m_ ratios) of the corresponding PpAAT1-SMs. However, substitutions of all 11 candidate amino acid residues dramatically increased the *K*_m_ values and decreased the *K*_cat_/*K*_m_ ratios of the corresponding PpAAT1-SMs, especially the substitutions of H165 and D376, which led to undetectable *K*_m_ values and *K*_cat_/*K*_m_ ratios of the site-directed mutant proteins (Table 2).

**Table 2:** Enzyme kinetics of PpAAT1 and its site-directed mutant proteins (PpAAT1-SMs) using 4-hydroxy-decanoyl–CoA as substrate

Enzyme	*K*_m_ (mM) (±SE)	*K*_cat_ (S^−1^) (±SE)	*K*_cat_/*K*_m_ (±SE)
PpAAT1	2.6187 (±0.2511)^c^	2.7216 (±0.1458)^a^	1.0392 (±0.0916)^a^
H165 V	N.D.	N.D.	N.D.
A170S	4.5852 (±0.6923)^b^	1.2770 (±0.1002)^b^	0.2785 (±0.0194)^b^
A174S	9.1599 (±1.0952)^a^	0.3992 (±0.0480)^c^	0.0436 (±0.0069)^c^
L117T	4.6670 (±0.8953)^b^	1.0455 (±0.0936)^b^	0.2240 (±0.0386)^b^
S262A	2.9920 (±0.3836)^c^	2.3630 (±0.3639)^a^	0.7898 (±0.1899)^a^
F264Y	3.0241 (±0.5145)^c^	2.5268 (±0.2867)^a^	0.8356 (±0.1217)^a^
K298F	10.1014 (±1.4928)^a^	0.4507 (±0.0624)^c^	0.0441 (±0.0079)^c^
F314Y	4.2018 (±0.5997)^b^	1.1131 (±0.1193)^b^	0.2649 (±0.0311)^b^
R339 V	8.8556 (±1.4954)^a^	0.3218 (±0.0547)^c^	0.0363 (±0.0051)^c^
K342F	5.0532 (±0.3364)^b^	1.3230 (±0.0927)^b^	0.2618 (±0.0742)^b^
S375A	2.8844 (±0.3293)^c^	2.3007 (±0.3411)^a^	0.7976 (±0.1183)^a^
D376A	N.D.	N.D.	N.D.
H379 V	8.2152 (±0.9510)^a^	0.3620 (±0.0249)^c^	0.0441 (±0.0036)^c^
F382Y	9.0224 (±0.7366)^a^	0.3730 (±0.0138)^c^	0.0413 (±0.0027)^c^

The in vitro enzyme activities of PpAAT1 and its site-directed mutant proteins (PpAAT1-SMs) purified from *E. coli* were examined using 4-hydroxy-decanoyl–CoA as the substrate. Data are presented as the means ± SE. Letters indicate significant differences at *P* ≤ 0.05 (*n* = 3). *N.D.* not detectable

The functions of the 9 candidate amino acid residues possibly involved in esterification were also examined using, as substrates, acetyl–CoA and four different alcohols (propanol, hexanol, benzyl alcohol, and decanol), which could potentially be converted to ester compounds such as propyl acetate, hexyl acetate, decyl acetate, and phenylmethyl acetate, respectively. Among the 9 candidate amino acid residues, substitution of F43 did not cause any significant effects on the *K*_cat_/*K*_m_ ratio of the site-directed mutant protein (Table S1). However, substitutions of all the other 8 candidate amino acid residues dramatically decreased the *K*_cat_/*K*_m_ ratios of the site-directed mutant proteins, especially the substitution of H165, which led to an undetectable *K*_cat_/*K*_m_ ratio of the site-directed mutant protein (Table S1). Consistent with the MM-GBSA results in which the interaction energy of the PpAAT1–hexanol–acetyl–CoA complex was more stable than that of the PpAAT1–propanol–acetyl–CoA, PpAAT1–benzyl alcohol–acetyl–CoA, and PpAAT1–decanol–acetyl–CoA complexes, a higher *K*_cat_/*K*_m_ ratio was observed in all substitutions when hexanol was used as the substrate (Table S1).

### Substitution of key amino acid residues in PpAAT1 leads to decreased conversion efficiency of 4-hydroxydecanoyl–CoA to γ-decalactone in transgenic tobacco

To examine the in vivo enzyme activity of PpAAT1 and its site-directed mutant proteins (PpAAT1-SMs), *PpAAT1* and *PpAAT1*-*SM*s were transiently expressed in the leaves of tobacco (*Nicotiana benthamiana*). Enzymatic activity assays were performed by monitoring the contents of γ-decalactone using 4-hydroxydecanoyl–CoA as the substrate. Although it is difficult to evaluate the esterification catalytic activity due to the interference of endogenous alcohols and CoAs in *N. benthamiana*^16^, the activities of PpAAT1 and PpAAT1-SMs possibly involved in internal esterification were verified. Consistent with the results of in vitro enzyme activity analyses, among the 14 candidate amino acid residues possibly involved in the internal esterification reaction, substitutions of S262, F264, and S375 did not cause any significant effects on the production of γ-decalactone in the leaves of transgenic tobacco expressing the corresponding site-directed mutant proteins. However, substitutions of all 11 candidate amino acid residues dramatically decreased the production of γ-decalactone, especially the substitutions of H165 and D376, which led to γ-decalactone being completely undetectable in the leaves of transgenic tobacco (Fig. 3b).

### Substitution of key amino acid residues in PpAAT1 disturbs the restoration of γ-decalactone and ester production in low-aroma peach fruits

To further investigate the contributions of the candidate amino acid residues to the internal esterification and its reactions of PpAAT1 in vivo, pMDC32-HPB-*PpAAT1*, or each of the 14 pMDC32-HPB-*PpAAT1*-*SMs* for the internal esterification reaction and the 9 pMDC32-HPB-*PpAAT1*-*SMs* for the esterification reaction, was injected into ‘Shazhi’ fruits. The site mutations of Ile49 to Val and Ala343 to Val of PpAAT1 in this cultivar led to undetectable γ-decalactone production and decreased ester accumulaton^14^. We found that the expression of *PpAAT1* and *PpAAT1-SMs*, except with the H165 and D376 mutations, partially restored the production of γ-decalactone and esters (Table S2). The levels of γ-decalactone and ester compounds in the injected “Shazhi” fruits were significantly higher than those in the untransformed wild-type fruits. Correspondingly, the contents of 4-hydroxydecanoyl–CoA, hexanol, benzyl alcohol, decanol, and propanol in the injected fruits decreased significantly. Further consumer panel evaluation demonstrated that injected “Shazhi” fruits produced an increased aroma/flavor, giving about the same smell as that of the high-aroma cultivar “Fenghuayulu” (Table S3).

### Substitution of key amino acid residues in PpAAT1 from different peach cultivars is closely related to fruit aroma intensity

Peach fruit aroma intensity is classified into three levels: strong, intermediate, and slight^21^. To further confirm the correlation of amino acid substitution and aroma production in peach fruits, we isolated *PpAAT1* from different cultivars. A total of 17 peach cultivars grown in the YanTai Germplasm Repository (Yantai, Shandong Province, China) and assessed as high-, middle-, or low-aroma peach varieties over five years were selected, and the amino acid sequences of PpAAT1 were analyzed. Consistent with our results, different amino acid substitutions were identified in the proteins encoded by *PpAAT1* isolated from all the middle- and low-aroma cultivars, whereas no amino acid substitution was found in the proteins encoded by *PpAAT1* isolated from high-aroma cultivars (Fig. 3c; Fig. S1). In PpAAT1 from low-aroma cultivars I and II, two amino acid substitutions (I49 V and A343 V, A343 V and H379 V) occurred. In PpAAT1 from the middle-aroma cultivars I–III, one (F382 V), two (T20S and K342R), and two (Q38P and L177F) amino acid substitutions occurred, respectively. Among amino acid residues, except for the substitutions of T20S and Q38P in some middle-aroma cultivars, all the substituted amino acids were also candidate key amino acid residues examined here and in our previous study^14^.

## Discussion

Protein homology modeling combined with site-directed mutagenesis has been used as an effective approach to unveil the catalytic mechanisms of different enzymes in various plant species. Using this strategy, the conserved amino acid residue critical for both product and substrate specificity in triterpene synthase has been successfully identified^22^. The key active site residues controlling the specificity for different 2-oxo substrates in methylthioalkylmalate synthase were also verified^23^. Based on computer modeling and molecular docking analyses, 16 candidate amino acid residues involved in the internal esterification reaction and 9 candidate amino acid residues involved in the esterification reaction of PpAAT1 were predicted (Fig. 1a, d–g). Further RMSD and MM-GBSA analyses showed that PpAAT1–ligand complexes reached stability after 30 ns during MDS assays, and the vdW values made the greatest contribution to the total free energy (Table 1; Fig. 2a–d). Similar results were also observed in the identification of the key residues of lipid transfer proteins (LTPs) in *Lotus japonicus*^24^. To date, studies on the functions of alcohol acyltransferases in plants have mainly focused on their esterification activity^7,25–27^. We previously reported that, like other acyltransferases that catalyze the esterification reaction, PpAAT1 in peach can also catalyze the internal esterification reaction^14^. Here, the candidate amino acid residues possibly contributing to the enzyme activity in PpAAT1 were clarified in vitro and in vivo.

In vitro enzymatic assays have been used as an indispensable part of the enzymatic characterization of AATs in the fruits of different plant species, such as strawberry, apple, and papaya, in which alcohol acyltransferases catalyze the conversion of a series of alcohols and coenzyme A to esters^7,16,28^. To test the internal esterification and its activities of PpAAT1 in vitro, *PpAAT1* and *PpAAT1-SMs* were heterologously expressed in *E. coli*, and the activities of PpAAT1 and PpAAT1-SMs using 4-hydroxydecanoyl–CoA as substrates for the internal esterification reaction and acetyl–CoA and alcohols (propanol, benzyl alcohol, and decanol) as substrates for the esterification reaction were examined. The substitutions of the key amino acid residues possibly involved in both reactions dramatically decreased the enzymatic activities of PpAAT1-SMs (Table 2 and Table S1). Both His165 in the HxxxD motif and D376 in the nonconservative region play a catalytic role in the internal esterification reaction. In the mountain papaya alcohol acyltransferase (VpAAT1), mutation of a noncatalytic residue D381 in the conserved DF(V)GWG motif led to a dramatic reduction in the binding capacity to several substrates in the esterification reaction^29^. In PpAAT1, D376 does not belong to the DF(V)GWG motif, indicating that the processes of internal esterification and esterification of AATs could be different. Among the 11 key amino acid residues involved in the internal esterification reaction, four of them (H165, A174, K298, and F314) were also involved in the esterification reaction. However, among the eight key amino acid residues involved in the esterification reaction, only H165 and F314, which were also involved in the internal esterification reaction, were involved in the internal esterification reaction, while the remaining six key amino acid residues were not (Table S4).

Tobacco has been used as a model plant for the functional verification of plant enzymes. To test the enzymatic activities of PpAAT1 and PpAAT1-SMs in vivo, we performed enzyme activity analyses in both tobacco leaves and peach fruits. To date, no lactones derived from hydroxy fatty acids have been reported in tobacco. Since endogenous alcohols and coenzyme A may influence the esterification activity of exogenous AATs^16^, we examined the internal esterification activity of PpAAT1 and PpAAT1-SMs in the leaves of transgenic tobacco. The contents of γ-decalactone produced in the leaves transformed with *PpAAT1* and different *PpAAT1-SM*s were compared. Consistent with the in vitro enzymatic activities, the production of γ-decalactone in transgenic tobacco leaves expressing *PpAAT1-SM*s with mutations in key amino acid residues was markedly reduced (Fig. 3b). Similar results were also observed in transgenic tobacco expressing MpAAT1 and CYP76F14^16,30^. To further test the functions of PpAAT1 and PpAAT1-SMs in peach fruits, *PpAAT1* and *PpAAT1-SM*s were transiently expressed in the fruits of the low-aroma Shazhi cultivar. Expression of *PpAAT1* and *PpAAT1-SMs* only partially restored the biosynthesis of γ-decalactone and esters in transgenic peach fruits, especially the PpAAT1-SMs with the mutations of H165 and D376, which failed to restore the biosynthesis of γ-decalactone and esters (Table S2). Similarly, replacement of the conserved His or Asp in the HxxxD motif of vinorine synthase resulted in complete loss of enzyme activity^19^. Therefore, both H165 and D376 play a crucial role in maintaining the enzymatic activity of PpAAT1.

The activity of AAT1 is critical for ester production, especially in low-aroma cultivars. In apple plants, phenotypic variability in ester content was observed in different varieties with different fruit aromas^25^. The enzymatic activity of AAT1 in *Solanum lycopersicum*, which has a low ester volatile content, is relatively lower than that of AAT1 in *Solanum pennellii*, which has a high ester volatile content^26^. We compared the amino acid sequence differences of PpAAT1 from different cultivars producing low-, middle-, and high-level fruit aromas. Consistent with previous reports, different amino acid substitutions were identified in both low- and middle-aroma species, implying a close relationship between key amino acid substitution, AAT1 activity, and ester production (Fig. 3c).

**Fig. 4:**
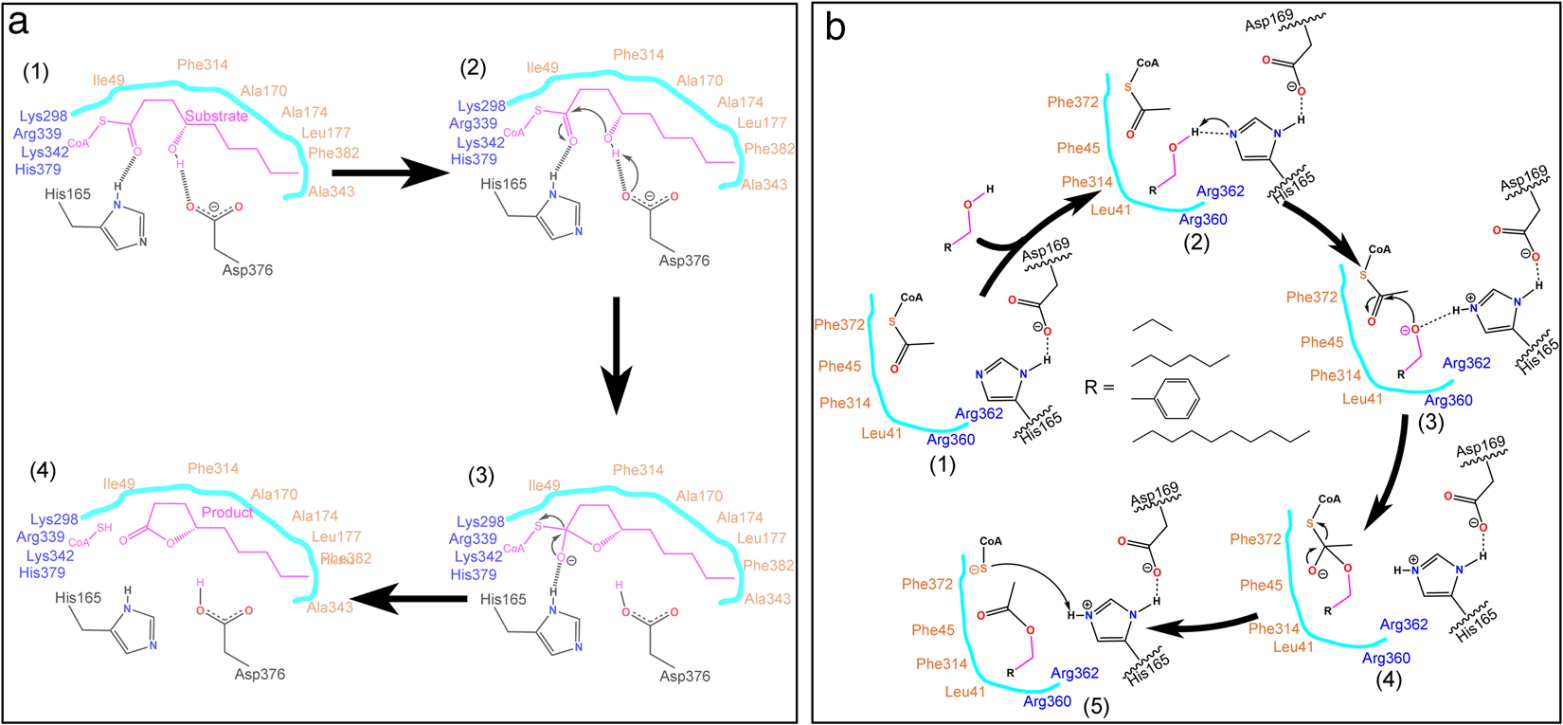
Proposed catalytic mechanism of PpAAT1. **a** Internal esterification reaction using 4-hydroxydecanoyl–CoA as the substrate. (1) His165 is oriented to the carbonyl group, while Asp376 is oriented to the hydroxyl group of 4-hydroxydecanoyl–CoA, (2) the hydrogen atom of the hydroxyl group of 4-hydroxydecanoyl–CoA is transferred to the carboxyl group of Asp376 to form an aromatic nucleus, (3) the carbon–oxygen double bond is formed, and the CoA-SH group is removed from 4-hydroxydecanoyl–CoA, and (4) γ-decalactone is formed. **b** Esterification reaction using acetyl–CoA and alcohols as substrates. (1) substrates at the catalytic center, (2) the hydroxyl group of alcohols forms a hydrogen bond with His165, (3) a hydrogen atom is transferred to a N atom on His165 and the alcohol substrate forms an O anion to attack the acetyl C atom of acetyl–CoA, (4) the substrates form a C=O double bond and the negatively charged coenzyme A, and (5) an ester is formed and the negatively charged coenzyme A accepts a hydrogen atom from His165 to form coenzyme A

Taken together, our findings in the present study indicate that both residues H165 and D376 are indispensable for the internal esterification reaction, whereas only H165 is required for the esterification reaction in PpAAT1. During the internal esterification reaction of PpAAT1, both residues H165 and D376 directly interact with the substrate, and the other 11 amino acid residues function in substrate recognition and spatial conformation in the reaction center. Initially, His165 is oriented to the carbonyl group, while Asp376 is oriented to the hydroxyl group. Then, the hydrogen atom on the hydroxyl group of the substrate is transferred to the carboxyl group of Asp376 to form an aromatic nucleus. Finally, the CoA-SH group is removed to form the final lactone products (Fig. 4a). During the esterification reaction of PpAAT1, the hydroxyl group forms a hydrogen bond with His165, which then attacks the acetyl C atom of acetyl–CoA to form an ester (Fig. 4b).

## Materials and methods

### Plant materials

Fruits of the peach cultivars “Fenghuayulu” and “Shazhi” were harvested from the Germplasm Resource Nursery of Yantai Academy of Agricultural Sciences (Yantai, China). “Fenghuayulu” is an heirloom Chinese peach cultivar that possesses a strong aroma. “Shazhi” is a modern cultivar that possesses a very weak aroma. The aromas of these cultivars have been consistently recorded over 5 years during cultivation in the nursery. Other peach cultivars used in this study were also collected from the Germplasm Resource Nursery of Yantai Academy of Agricultural Sciences.

To ensure that all the fruit samples were at the same level of maturity, the index of the absorbance difference (*I*_AD_) was used as described previously^31^. “Fenghuayulu” fruits were sampled 123 days after full bloom (DAFB) (*I*_AD_ = 0.1), which is the optimal stage of maturity for harvest and sale. The sampling points for “Shazhi” fruits correspond to the same fruit maturity stage as that used for “Fenghuayulu” based on *I*_AD_, with the fruits collected at 110 DAFB (*I*_AD_ = 0.1). Three biological replicates, with ten fruits in each, were sampled in this study.

### PpAAT1 gene isolation and molecular docking analysis

Total RNA was extracted from the fruit flesh of “Fenghuyulu” using a TaKaRa MiniBEST Plant RNA Extraction Kit and treated with RNase-free DNase I (Takara, Dalian, China). RNA quantity and quality were evaluated using a NanoPhotometer spectrophotometer (Implen, Munich, Germany). First-strand cDNA was synthesized using a PrimeScript II First Strand cDNA Synthesis Kit (Takara) according to the manufacturer’s instructions. Primer sequences for cloning the CDS of *PpAAT1* and PCR conditions were the same as those described previously^14^. PCR products were sequenced at Sangon Biotech (Shanghai, China) Co., Ltd.

Modeler v9.19 (http://salilab.org/modeller/) was used for homology modeling of the PpAAT1 protein, with the crystal structure of an Arabidopsis (*Arabidopsis thaliana*) acyltransferase protein (Protein Data Bank no. 5KJT) as template, and the protein was subjected to energy minimization treatment and served as a receptor structure for molecular docking. To evaluate the stereochemical quality of the PpAAT1 3D model, Ramachandran’s map was generated using PROCHECK^32^. Verify3D was used to analyze the compatibility of the PpAAT1 model with its own amino acid sequence^33^. The structures of the substrates (propanol, hexanol, benzyl alcohol, and decanol) were constructed with acetyl–CoA using AutoDock4.2.6^34^ and optimized using the MOPAC program^35^. To exclude the unreasonable spatial structure and make the binding model more stable, energy optimization was performed using an Amber14 force field^36^. Energy minimization was completed by two steps. First, 2000 steps of the steepest descent method were performed, followed by another 2000 steps of the conjugate gradient method. The optimized structure was used for further analysis. MDSs of the substrates of PpAAT1 were performed using the Amber 18 Software package together with the TIP3P model for water^37,38^. The initial coordinates for the MD calculations were taken from the docking experiments. The MD simulation process was carried out in two steps: first, a 2-ns constrained solute MD simulation was carried out, and the system temperature was gradually increased from 0 to 300 K; then, a 40-ns MD simulation was carried out. During MD simulation, motion equations were integrated with a 2-fs time step, and the data were collected every 10 ps. Visualization of PpAAT1–ligand complexes and MD trajectory analysis were carried out using the VMD software package^39^. The MM/GBSA method was used to estimate the binding free energies of the PpAAT1–ligand complexes^24,29,40,41^.

### Site-directed mutation and heterologous expression of PpAAT1 proteins in *E. coli*

To better clarify the catalytic activities of the key amino acid residues of the PpAAT1 protein, each of the predicted candidate amino acid residues was substituted with a corresponding amino acid with opposite polarity^42^. In this way, the 14 candidate amino acid residues His165, Ala170, Ala174, Leu177, Ser262, Phe264, Lys298, Phe314, Arg339, Lys342, Ser375, Asp376, His379, and Phe382 were replaced with Val, Ser, Ser, Thr, Ala, Tyr, Phe, Tyr, Val, Phe, Ala, Ala, Val, and Tyr for the internal esterification analyses, and the 9 candidate amino acid residues Leu41, Phe43, Phe45, His165, Asp169, Phe314, Arg360, Arg362, and Phe372 were replaced with Thr, Thr, Thr, Val, Ala, Tyr, Val, Val, and Tyr for the esterification analyses, respectively. A C-terminal polyhistidine tag was included in both *PpAAT1* and its site-directed mutant forms (*PpAAT1*-*SMs*). For enzyme activity assays, sequence codons were optimized and synthesized at GenScript Co. Ltd. (Nanjing, China). Then, the optimized sequences were cloned into the PET-30a (+) vector for expression in *E. coli* cells. Heterologous expression, purification, and identification of the recombinant proteins (PpAAT1 and PpAAT1-SMs) were conducted at GenScript Co., Ltd. (Nanjing, China).

### Enzyme activity assays

The enzyme activities of PpAAT1 and PpAAT1-SMs for γ-decalactone formation using 4-hydroxy-decanoyl–CoA (Siruike Co. Ltd., NanJing, China) as substrate were assessed via GC–MS analyses as described previously^14^. For esterification assays, propanol, hexanol, benzyl alcohol, decanol, and acetyl–CoA (Sigma-Aldrich, USA) were used as substrates to produce the esters found in peach fruits. Qualitative and quantitative determination of 4-hydroxy-decanoyl–CoA was performed at Siruike Co., Ltd. Boiled proteins were used as a control sample. All experiments were performed in triplicate. The turnover number (*k*_cat_) and affinity (*K*_m_) were calculated using Origin 2018 (www.originlab.com).

### Construction of plant expression vectors

To assess the enzymatic activity of PpAAT1 and PpAAT1-SMs in plants, *PpAAT1* isolated from ‘Fenghuayulu’ and its mutant forms (*PpAAT1*-*SMs*) synthesized at GenScript Co. Ltd. (Nanjing, China) were cloned into pMDC32-HPB (Addgene: 32078), driven by the cauliflower mosaic virus 35S promoter, to generate the plant expression vectors pMDC32-HPB-*PpAAT1* and pMDC32-HPB-*PpAAT1*-*SMs*.

### Transient expression of PpAAT1 and PpAAT1-SM proteins in tobacco leaves

To assess the internal esterification activity of PpAAT1 and PpAAT1-SMs in tobacco, the GV3101 (pSoup-P19) (Shanghai Weidi Biotechnology Co., Ltd) strain containing pMDC32-HPB-*PpAAT1* or each of the 14 pMDC32-HPB-*PpAAT1*-*SMs* was infiltrated into tobacco leaves, together with the substrate of 4-hydroxy-decanoyl–CoA (10 mM), as described previously^14^. GC–MS analyses were carried out to quantify γ-decalactone production as described previously^14^.

### Overexpression of PpAAT1 and PpAAT1-SMs in peach fruits

For the overexpression of PpAAT1 and PpAAT1-SMs, the GV3101 (pSoup-P19) strain containing pMDC32-HPB-*PpAAT1* or each of the 14 pMDC32-HPB-*PpAAT1*-*SMs* for the internal esterification reaction and the 9 pMDC32-HPB-*PpAAT1*-*SMs* for the esterification reaction was injected into “Shazhi” fruits at 106 DAFB, and the contents of γ-decalactone and esters were determined via GC–MS analyses as described previously once the transgenic fruits reached their optimal harvesting stage for sale (evaluated by *I*_AD_)^14^.

#### Statistical analyses

All experiments were conducted in triplicate. IBM SPSS Statistics 23 was used to analyze the significant differences, and comparisons were analyzed by Fisher’s LSD test (*P* ≤ 0.05). Histograms were prepared with Origin 2018. The amino acid sequences for PpAAT1, derived from all kinds of peach cultivars, were phylogenetically analyzed using the maximum likelihood method with 1000 bootstrap replicates using MEGA-X.

## Supplementary information

Supplemental materials

## Supplementary information

**Supplementary Information** The online version contains supplementary material available at (10.1038/s41438-021-00461-x).
