# Supplementary material for: Site-directed mutagenesis identified the key active site residues of alcohol acyltransferase PpAAT1 responsible for aroma biosynthesis in peach fruits
Source: Hortic Res. 2021 Feb 1;8:32. doi: 10.1038/s41438-021-00461-x (PMC7847995; doi:10.1038/s41438-021-00461-x)
Supplement: Supplementary file 1 — Supplemental materials [file 41438_2021_461_MOESM1_ESM.docx]

**Title:**

Site-directed mutagenesis identified the key active site residues of alcohol acyltransferase PpAAT1 responsible for aroma biosynthesis in peach fruits

**Running title:**

Active site residues of alcohol acyltransferase

**Authors:**

Zhi-Zhong Song^1,2,#^, Bin Peng^1,2,#,*^, Zi-Xia Gu^3,#^, Mei-Ling Tang^2,4^, Bei Li^1,2^, Mei-Xia Liang^1,2^, Li-Min Wang^1,2^, Xiao-Tong Guo^1,2^, Jian-Ping Wang^4^, Yu-Fen Sha^4^, Hong-Xia Zhang^1,2,*^

***Corresponding authors:**

Bin Peng: bingo1937@foxmail.com

Hong-Xia Zhang: 3213@ldu.edu.cn; hxzhang@sibs.ac.cn

Phone: 86-0535-6664662; Fax: 86-0535-6664660

^#^ These authors contributed equally to this work.

**Table S1** Enzyme kinetics of PpAAT1 and its site-directed mutant proteins (PpAAT1-SMs) using acetyl-CoA and different alcohols as substrates

| Enzymes | Propanol | | | Hexanol | | | Benzyl alcohol | | | Decanol | | |
| --- | --- | --- | --- | --- | --- | --- | --- | --- | --- | --- | --- | --- |
|  | *K*_m_ (mM) (±SE) | *K*_cat_ (S^-1^) (±SE) | *K*_cat_/*K*_m_ (±SE) | *K*_m_ (mM) (±SE) | *K*_cat_ (S^-1^) (±SE) | *K*_cat_/*K*_m_ (±SE) | *K*_m_ (mM) (±SE) | *K*_cat_ (S^-1^) (±SE) | *K*_cat_/*K*_m_ (±SE) | *K*_m_ (mM) (±SE) | *K*_cat_ (S^-1^) (±SE) | *K*_cat_/*K*_m_ (±SE) |
| PpAAT1 | 5.1552 (±0.1588)^d^ | 2.2345 (±0.1033)^a^ | 0.4336 (±0.0288)^a^ | 3.8314 (±0.2612)^d^ | 6.1041 (±0.4223)^a^ | 1.5811 (±0.0970)^a^ | 4.3682 (±0.2733)^d^ | 2.2453 (±0.1868)^a^ | 0.5132 (±0.0511)^a^ | 8.5053 (±0.9422)^d^ | 0.9436 (±0.0842)^a^ | 0.1106 (±0.0104)^a^ |
| L41T | 6.7621 (±0.4162)^c^ | 1.1650 (±0.0912)^b^ | 0.1622 (±0.0151)^d^ | 5.2100 (±0.5052)^c^ | 3.2833 (±0.1449)^c^ | 0.6210 (±0.0359)^c^ | 10.1670 (±1.1426)^c^ | 1.1456 (±0.1105)^c^ | 0.1075 (±0.0124)^b^ | 18.5860 (±2.0450)^a^ | 0.6173 (±0.0356)^b^ | 0.0310 (±0.0021)^c^ |
| F43T | 5.5856 (±0.3914)^d^ | 1.9619 (±0.1975)^a^ | 0.3411 (±0.0312)^b^ | 4.1400 (±0.5995)^cd^ | 5.4360 (±0.5014)^a^ | 1.3111 (±0.1861)^a^ | 4.9753 (±0.4332)^d^ | 1.9612 (±0.1742)^a^ | 0.4122 (±0.0562)^a^ | 8.7446 (±0.9624)^d^ | 0.9356 (±0.0803)^a^ | 0.1034 (±0.0089)^a^ |
| F45T | 7.2700 (±0.3233)^b^ | 0.8511 (±0.0960)^c^ | 0.1188 (±0.0163)^e^ | 4.3922 (±0.3899)^cd^ | 2.7590 (±0.1965)^d^ | 0.6215 (±0.0476)^c^ | 15.1478 (±1.0216)^a^ | 1.0398 (±0.0788)^c^ | 0.0682 (±0.0069)^c^ | 11.7123 (±0.5235)^b^ | 0.4633 (±0.0375)^c^ | 0.0383 (±0.0027)^b^ |
| H165V | N.D. | N.D. | N.D. | N.D. | N.D. | N.D. | N.D. | N.D. | N.D. | N.D. | N.D. | N.D. |
| D169A | 7.6551 (±0.2469)^b^ | 0.2411 (±0.0177)^d^ | 0.0324 (±0.0019)^f^ | 10.5945 (±0.8307)^a^ | 0.8705 (±0.0546)^f^ | 0.0821 (±0.0043)^f^ | 10.0889 (±0.7212)^c^ | 0.5556 (±0.2440)^d^ | 0.0529 (±0.0028)^d^ | 10.8331 (±0.2699)^c^ | 0.1103 (±0.0076)^f^ | 0.0101 (±0.0008)^e^ |
| F314Y | 8.8463 (±0.3546)^a^ | 0.9245 (±0.0586)^c^ | 0.1068 (±0.0057)^e^ | 6.7568 (±0.4996)^b^ | 2.5288 (±0.2106)^d^ | 0.3768 (±0.0289)^e^ | 14.7156 (±0.9852)^a^ | 1.0620 (±0.0344)^c^ | 0.0075 (±0.0006)^e^ | 8.8447 (±0.4034)^d^ | 0.3714 (±0.0142)^d^ | 0.0416 (±0.0039)^b^ |
| R360V | 6.4339 (±0.3008)^c^ | 0.7767 (±0.0817)^c^ | 0.1228 (±0.0101)^e^ | 7.1266 (±0.5669)^b^ | 2.1584 (±0.1044)^e^ | 0.3525 (±0.0309)^e^ | 10.1313 (±1.3467)^c^ | 0.7533 (±0.0574)^d^ | 0.0742 (±0.0059)^c^ | 10.2865 (±0.7423)^c^ | 0.1227 (±0.0109)^f^ | 0.0123 (±0.0017)^e^ |
| R362V | 6.1667 (±0.4325)^c^ | 1.3413 (±0.0982)^b^ | 0.2011 (±0.0159)^c^ | 4.6903 (±0.2411)^c^ | 4.2889 (±0.3044)^b^ | 0.9177 (±0.0448)^b^ | 13.1788 (±0.4143)^b^ | 1.4817 (±0.0917)^b^ | 0.1138 (±0.0103)^b^ | 13.1429 (±1.2519)^b^ | 0.5716 (±0.0442)^b^ | 0.0433 (±0.0037)^b^ |
| F372T | 7.5221 (±0.3133)^b^ | 0.8642 (±0.0836)^c^ | 0.1059 (±0.0105)^e^ | 4.8969 (±0.3653)^c^ | 2.3868 (±0.2688)^d^ | 0.4867 (±0.0221)^d^ | 16.0550 (±0.5090)^a^ | 0.9519 (±0.0917)^c^ | 0.0562 (±0.0054)^d^ | 12.1876 (±0.9921)^b^ | 0.2993 (±0.0300)^e^ | 0.0254 (±0.0018)^d^ |

The *in vitro* enzyme activities of PpAAT1 and its site-directed mutant proteins (PpAAT1-SMs) purified from *E. Coli.* using acetyl-CoA and different alcohols as substrates were examined. Data are presented as means±SE. Letters indicate significant difference at *P* ≤ 0.05 (n = 3). N.D., not detectable.

**Table S2** γ-Decalactone, ester, alcohol and 4-hydroxydecanoyl-CoA contents in peach fruits expressing PpAAT1 and its site-directed mutant proteins (PpAAT1-SMs)

| Genotype | γ-Decalactone | 4-Hydroxydecanoyl-coA | Decyl acetate | Phenylmethyl acetate | Hexyl acetate | Propyl acetate | Hexanol | Benzyl alcohol | propanol | Decanol |
| --- | --- | --- | --- | --- | --- | --- | --- | --- | --- | --- |
| ‘Fenghuayulu’ | 2.9349 (±0.2057)^a^ | 4.6318 (±0.5821)^a^ | 0.1958 (±0.0146)^a^ | 0.2113 (±0.0185)^a^ | 1.1458 (±0.1009)^a^ | 2.3019 (±0.1980)^a^ | 0.8554 (±0.0769)^c^ | 6.514 (±0.5526)^a^ | 5.9516  (±0.3846)^a^ | 1.1381 (±0.0955)^b^ |
| ‘Shazhi’ | N.D. | 5.1582 (±0.8121)^a^ | N.D. | N.D. | 0.0976 (±0.0103)^d^ | 0.6941 (±0.0922)^d^ | 1.6839 (±0.1992)^a^ | 1.1642 (±0.1096)^b^ | 3.8799 (±0.2177)^b^ | 1.5326 (±0.1716)^a^ |
| PpAAT1 | 0.5243 (±0.1882)^b^ | 1.0282 (±0.2114)^d^ | 0.0855 (±0.0198)^b^ | 0.1047 (±0.0326)^b^ | 0.5874 (±0.1792)^b^ | 1.6216 (±0.3062)^b^ | 0.4219 (±0.1063)^d^ | 0.3125 (±0.0701)^e^ | 2.0016 (±0.3383)^d^ | 0.6324 (±0.1084)^c^ |
| H165V | N.D. | 4.9569 (±0.7211)^a^ | N.D. | N.D. | 0.1095 (±0.0106)^d^ | 0.7112 (±0.1003)^d^ | 1.5716 (±0.1986)^a^ | 1.1381 (±0.1432)^b^ | 3.4668 (±0.4681)^b^ | 1.5226 (±0.1716)^a^ |
| A170S | 0.2019 (±0.0732)^c^ | 2.2171 (±0.2917)^c^ | 0.0796 (±0.0117)^b^ | 0.0890 (±0.0288)^b^ | 0.5417 (±0.1406)^b^ | 1.5814 (±0.2647)^b^ | 0.4653 (±0.1129)^d^ | 0.3817 (±0.0662)^e^ | 2.1316 (±0.3068)^d^ | 1.5139 (±0.1611)^a^ |
| A174S | 0.0674 (±0.0161)^de^ | 3.5685 (±0.5126)^ab^ | 0.0513 (±0.0078)^c^ | 0.0355 (±0.0071)^c^ | 0.2542 (±0.0411)^c^ | 1.0321 (±0.1136)^c^ | 0.8816 (±0.1141)^c^ | 0.5628 (±0.0794)^d^ | 2.6345 (±0.3655)^cd^ | 1.0141 (±0.1547)^b^ |
| L117T | 0.2228 (±0.0692)^c^ | 2.1668 (±0.3155)^c^ | 0.0813 (±0.0186)^b^ | 0.1009 (±0.0364)^b^ | 0.5629 (±0.1577)^b^ | 1.6041 (±0.2251)^b^ | 0.4776 (±0.1034)^d^ | 0.3645 (±0.0702)^e^ | 2.0985 (±0.3112)^d^ | 1.5197 (±0.1813)^a^ |
| S262A | 0.4335 (±0.1885)^b^ | 1.0113 (±0.2002)^d^ | 0.0786 (±0.0159)^b^ | 0.0904 (±0.0294)^b^ | 0.5443 (±0.1619)^b^ | 1.5916 (±0.2714)^b^ | 0.4656 (±0.1055)^d^ | 0.3881 (±0.0831)^e^ | 2.1023 (±0.2974)^d^ | 1.5154 (±0.1745)^a^ |
| F264Y | 0.4673 (±0.1945)^b^ | 1.0046 (±0.1889)^d^ | 0.0764 (±0.0126)^b^ | 0.0931 (±0.0315)^b^ | 0.5342 (±0.1796)^b^ | 1.6117 (±0.2842)^b^ | 0.4834 (±0.1011)^d^ | 0.3573 (±0.0696)^e^ | 2.0885 (±0.2855)^d^ | 1.5201 (±0.1831)^a^ |
| K298F | 0.0851 (±0.0205)^d^ | 3.4322 (±0.4008)^b^ | 0.0521 (±0.0071)^c^ | 0.0372 (±0.0061)^c^ | 0.2491 (±0.0392)^c^ | 1.0041 (±0.1016)^c^ | 0.8245 (±0.1081)^c^ | 0.6199 (±0.0894)^d^ | 2.6155 (±0.3195)^cd^ | 1.0156 (±0.1482)^b^ |
| F314Y | 0.1884 (±0.0604)^c^ | 2.3461 (±0.3017)^c^ | 0.0328 (±0.0081)^d^ | 0.0346 (±0.0086)^c^ | 0.2002 (±0.0456)^c^ | 1.0551 (±0.1136)^c^ | 0.8619 (±0.0919)^c^ | 0.5777 (±0.0884)^d^ | 2.5931 (±0.2874)^cd^ | 1.0206 (±0.1519)^b^ |
| R339V | 0.0452 (±0.0131)^e^ | 3.9443 (±0.3465)^ab^ | 0.0759 (±0.0166)^b^ | 0.0893 (±0.0200)^b^ | 0.5422 (±0.1647)^b^ | 1.5526 (±0.2411)^b^ | 0.4631 (±0.0954)^d^ | 0.3644 (±0.0582)^e^ | 2.1136 (±0.3022)^d^ | 1.5174 (±0.1796)^a^ |
| K342F | 0.2118 (±0.0662)^c^ | 2.2140 (±0.3155)^c^ | 0.0784 (±0.0179)^b^ | 0.0911 (±0.0184)^b^ | 0.5586 (±0.1411)^b^ | 1.6001 (±0.2578)^b^ | 0.5008 (±0.1010)^d^ | 0.3821 (±0.0610)^e^ | 2.0259 (±0.3181)^d^ | 1.5205 (±0.1916)^a^ |
| S375A | 0.4116 (±0.1488)^b^ | 1.0206 (±0.1899)^d^ | 0.0810 (±0.0148)^b^ | 0.0915 (±0.0184)^b^ | 0.5284 (±0.1291)^b^ | 1.5763 (±0.2441)^b^ | 0.4762 (±0.1060)^d^ | 0.3254 (±0.0559)^e^ | 2.0654 (±0.2977)^d^ | 1.5099 (±0.1852)^a^ |
| D376A | N.D. | 5.0155 (±1.0306)^a^ | 0.0809 (±0.0135)^b^ | 0.0926 (±0.0179)^b^ | 0.5330 (±0.1342)^b^ | 1.6078 (±0.2355)^b^ | 0.4318 (±0.0886)^d^ | 0.3368 (±0.0605)^e^ | 2.1846 (±0.3019)^d^ | 1.5301 (±0.1926)^a^ |
| H379V | 0.0518 (±0.0128)^e^ | 4.0664 (±1.0021)^ab^ | 0.0836 (±0.0162)^b^ | 0.1002 (±0.0261)^b^ | 0.5664 (±0.1419)^b^ | 1.5378 (±0.2210)^b^ | 0.4472 (±0.0991)^d^ | 0.3511 (±0.0582)^e^ | 2.0941 (±0.2855)^d^ | 1.5321 (±0.2013)^a^ |
| F382Y | 0.0894 (±0.0201)^d^ | 3.5166 (±0.4133)^b^ | 0.0781 (±0.0182)^b^ | 0.1016 (±0.0249)^b^ | 0.5328 (±0.1617)^b^ | 1.5844 (±0.2051)^b^ | 0.4888 (±0.1049)^d^ | 0.3221 (±0.0637)^e^ | 2.2146 (±0.3211)^d^ | 1.5175 (±0.1826)^a^ |
| L41T | 0.4335 (±0.1678)^b^ | 5.0352 (±1.0011)^a^ | 0.0388 (±0.0076)^d^ | 0.0411 (±0.0062)^c^ | 0.2682 (±0.0402)^c^ | 1.0214 (±0.1311)^c^ | 0.8344 (±0.1003)^c^ | 0.5917 (±0.0836)^d^ | 2.7915 (±0.3401)^c^ | 1.0135 (±0.1816)^b^ |
| F43T | 0.4854 (±0.1622)^b^ | 4.8334 (±0.8009)^a^ | 0.0750 (±0.0112)^b^ | 0.0993 (±0.0291)^b^ | 0.5449 (±0.1619)^b^ | 1.5382 (±0.2788)^b^ | 0.4914 (±0.1121)^d^ | 0.3844 (±0.0579)^e^ | 1.9254 (±0.5321)^d^ | 0.6652 (±0.1431)^c^ |
| F45T | 0.4199 (±0.1711)^b^ | 4.9155 (±0.9100)^a^ | 0.0354 (±0.0063)^d^ | 0.0351 (±0.0076)^c^ | 0.2167 (±0.0419)^c^ | 1.0119 (±0.1271)^c^ | 0.7910 (±0.1016)^c^ | 0.5521 (±0.0762)^d^ | 2.5915 (±0.3382)^cd^ | 1.0127 (±0.1194)^b^ |
| D169A | 0.3825 (±0.1112)^b^ | 5.1114 (±0.9366)^a^ | 0.0139 (±0.0031)^e^ | 0.0103 (±0.0031)^d^ | 0.1121 (±0.0346)^d^ | 0.9021 (±0.1101)^cd^ | 1.1144 (±0.1021)^b^ | 0.8163 (±0.0852)^c^ | 2.9960 (±0.4012)^c^ | 1.0154 (±0.1210)^b^ |
| R360V | 0.4172 (±0.1517)^b^ | 5.1274 (±0.9786)^a^ | 0.0311 (±0.0069)^d^ | 0.0332 (±0.0054)^c^ | 0.2113 (±0.0421)^c^ | 1.0007 (±0.1293)^c^ | 0.7752 (±0.0952)^c^ | 0.5617 (±0.0691)^d^ | 2.5139 (±0.3394)^cd^ | 1.0097 (±0.1141)^b^ |
| R362V | 0.4555 (±0.1324)^b^ | 4.9352 (±0.8112)^a^ | 0.0533 (±0.0082)^c^ | 0.0381 (±0.0093)^c^ | 0.2613 (±0.0491)^c^ | 1.0316 (±0.1118)^c^ | 0.8567 (±0.1016)^c^ | 0.6018 (±0.0852)^d^ | 2.6045 (±0.3054)^c^ | 1.0236 (±0.1711)^b^ |
| F372T | 0.4015 (±0.1289)^b^ | 5.0864 (±0.9155)^a^ | 0.0371 (±0.0058)^d^ | 0.0332 (±0.0054)^c^ | 0.2120 (±0.0388)^c^ | 1.0245 (±0.1209)^c^ | 0.7660 (±0.0899)^c^ | 0.5483 (±0.0717)^d^ | 2.5668 (±0.3845)^cd^ | 1.0110 (±0.1365)^b^ |

The contents of γ-decalactone, ester, alcohol and 4-hydroxydecanoyl-CoA in the fruits of low-aroma cultivar ‘Shazhi’ expressing PpAAT1 and its site-directed mutant proteins (PpAAT1-SMs) were examined. The fruits of the high-aroma cultivar ‘Fenghuayulu’ and low-aroma cultivar ‘Shazhi’ were used as controls. Letters indicate significant difference at *P* ≤ 0.05 (n = 6). N.D., not detectable.

**Table S3** Consumer evaluation of the aroma intensity of peach fruits

| Genotype | Strong | Intermediate | Slight |
| --- | --- | --- | --- |
| PpAAT1 | 39 | 11 | 0 |
| H165V | 0 | 0 | 50 |
| A170S | 24 | 16 | 10 |
| A174S | 10 | 21 | 19 |
| L117T | 19 | 20 | 11 |
| S262A | 36 | 10 | 4 |
| F264Y | 33 | 12 | 5 |
| K298F | 12 | 29 | 9 |
| F314Y | 18 | 36 | 6 |
| R339V | 12 | 20 | 18 |
| K342F | 20 | 13 | 17 |
| S375A | 29 | 16 | 5 |
| D376A | 2 | 15 | 33 |
| H379V | 15 | 19 | 16 |
| F382Y | 26 | 11 | 13 |
| L41T | 6 | 30 | 14 |
| F43T | 11 | 34 | 5 |
| F45T | 9 | 28 | 13 |
| D169A | 5 | 18 | 27 |
| R360V | 7 | 29 | 14 |
| R362V | 2 | 41 | 7 |
| F372T | 2 | 36 | 12 |
| ‘Shazhi’ | 0 | 0 | 50 |

Fifty members of the consumer panels voted on the aroma intensity of wild type and transgenic ‘Shazhi’ fruits.

**Table S4** Enzyme efficiency analysis of PpAAT1 and its site-directed mutant proteins (PpAAT1-SMs)

| Enzyme | 4-hydroxy-decanoyl-CoA | Propanol | Hexanol | Benzyl alcohol | Decanol |
| --- | --- | --- | --- | --- | --- |
|  | *K*_cat_/*K*_m_ (±SE) | *K*_cat_/*K*_m_ (±SE) | *K*_cat_/*K*_m_ (±SE) | *K*_cat_/*K*_m_ (±SE) | *K*_cat_/*K*_m_ (±SE) |
| PpAAT1 | 1.0392(±0.0916)^a^ | 0.4336(±0.0288)^a^ | 1.5811(± 0.0970)^a^ | 0.5132(±0.0511)^a^ | 0.1106(±0.0104)^a^ |
| H165V | N.D. | N.D. | N.D. | N.D. | N.D. |
| A170S | 0.2785(±0.0194)^b^ | 0.4211(±0.0301)^a^ | 1.5219(± 0.1092)^a^ | 0.5008(±0.0520)^a^ | 0.1094(±0.0099)^a^ |
| A174S | 0.0436(±0.0069)^c^ | 0.3219(± 0.0287)^b^ | 0.6084(± 0.0233)^c^ | 0.1064 (± 0.0102)^b^ | 0.04071 (± 0.0029)^b^ |
| L117T | 0.2240(±0.0386)^b^ | 0.4186(±0.0334)^a^ | 1.5622(± 0.1355)^a^ | 0.5105(±0.0488)^a^ | 0.1052(±0.0108)^a^ |
| S262A | 0.7898(±0.1899)^a^ | 0.4327(±0.0285)^a^ | 1.5499(± 0.1018)^a^ | 0.5024(±0.0366)^a^ | 0.1044(±0.0089)^a^ |
| F264Y | 0.8356(±0.1217)^a^ | 0.4196(±0.0345)^a^ | 1.5687(± 0.1018)^a^ | 0.5024(±0.0366)^a^ | 0.1052(±0.0085)^a^ |
| K298F | 0.0441(±0.0079)^c^ | 0.3422(± 0.0309)^b^ | 0.5913(± 0.0271)^c^ | 0.1098 (± 0.0114)^b^ | 0.04176 (± 0.0038)^b^ |
| F314Y | 0.2649(±0.0311)^b^ | 0.1068(± 0.0057)^e^ | 0.3768( ± 0.0289)^e^ | 0.0075(± 0.0006)^d^ | 0.04162 (± 0.0039)^b^ |
| R339V | 0.0363(±0.0051)^c^ | 0.4267(±0.0228)^a^ | 1.5771(± 0.0820)^a^ | 0.5099(±0.0417)^a^ | 0.1066(±0.0093)^a^ |
| K342F | 0.2618(±0.0742)^b^ | 0.4055(±0.0305)^a^ | 1.5442(± 0.0913)^a^ | 0.5108(±0.0296)^a^ | 0.1023(±0.0084)^a^ |
| S375A | 0.7976(±0.1183)^a^ | 0.4115(±0.0295)^a^ | 1.5614(± 0.0824)^a^ | 0.5037(±0.0305)^a^ | 0.1102(±0.0111)^a^ |
| D376A | N.D. | 0.4207(±0.0331)^a^ | 1.5358(± 0.0914)^a^ | 0.5055(±0.0471)^a^ | 0.1060(±0.0139)^a^ |
| H379V | 0.0441(±0.0036)^c^ | 0.4118(±0.0402)^a^ | 1.5451(± 0.1130)^a^ | 0.5100(±0.0385)^a^ | 0.1044(±0.0112)^a^ |
| F382Y | 0.0413(±0.0027)^c^ | 0.4252(±0.0355)^a^ | 1.5500(± 0.1005)^a^ | 0.4918(±0.0410)^a^ | 0.1097(±0.0106)^a^ |
| L41T | 1.0114(±0.0826)^a^ | 0.1622 (±0.0151)^d^ | 0.6210 (±0.0359)^c^ | 0.1075(±0.0124)^b^ | 0.0310 (±0.0021)^c^ |
| F43T | 1.0274(±0.1003)^a^ | 0.3411(± 0.0312)^b^ | 1.3111 (±0.1861)^a^ | 0.4122(±0.0562)^a^ | 0.1034 (±0.0089)^a^ |
| F45T | 1.0315(±0.1014)^a^ | 0.1188(± 0.0163)^e^ | 0.6215 (±0.0476)^c^ | 0.0682(±0.0069)^c^ | 0.0383 (±0.0027)^b^ |
| D169A | 1.0099(±0.0917)^a^ | 0.0324 (±0.0019)^f^ | 0.0821 (±0.0043)^f^ | 0.0529 (±0.0028)^c^ | 0.0101 (±0.0008)^e^ |
| R360V | 1.0237(±0.0934)^a^ | 0.1228 (±0.0101)^e^ | 0.3525 (±0.0309)^e^ | 0.0742 (±0.0059)^c^ | 0.0123 (±0.0017)^e^ |
| R362V | 1.0057(±0.0884)^a^ | 0.2011 (±0.0159)^c^ | 0.9177 (±0.0448)^b^ | 0.1138 (±0.0103)^b^ | 0.0433 (±0.0037)^b^ |
| F372T | 1.0304(±0.1106)^a^ | 0.1059 (±0.0105)^e^ | 0.4867 (±0.0221)^d^ | 0.0562 (±0.0054)^d^ | 0.0254 (±0.0018)^d^ |

The *in vitro* enzyme efficiency of PpAAT1 and its site-directed mutant proteins (PpAAT1-SMs) purified from *E. Coli.* were examined using 4-hydroxy-decanoyl-CoA as substrate for internal-esterification reaction, and acetyl-CoA and different alcohols (propanol, hexanol, benzyl alcohol and decanol) as substrates for esterification reaction. Data are presented as means ± SE. Letters indicate significant difference at *P* ≤ 0.05 (n = 3). N.D., not detectable.


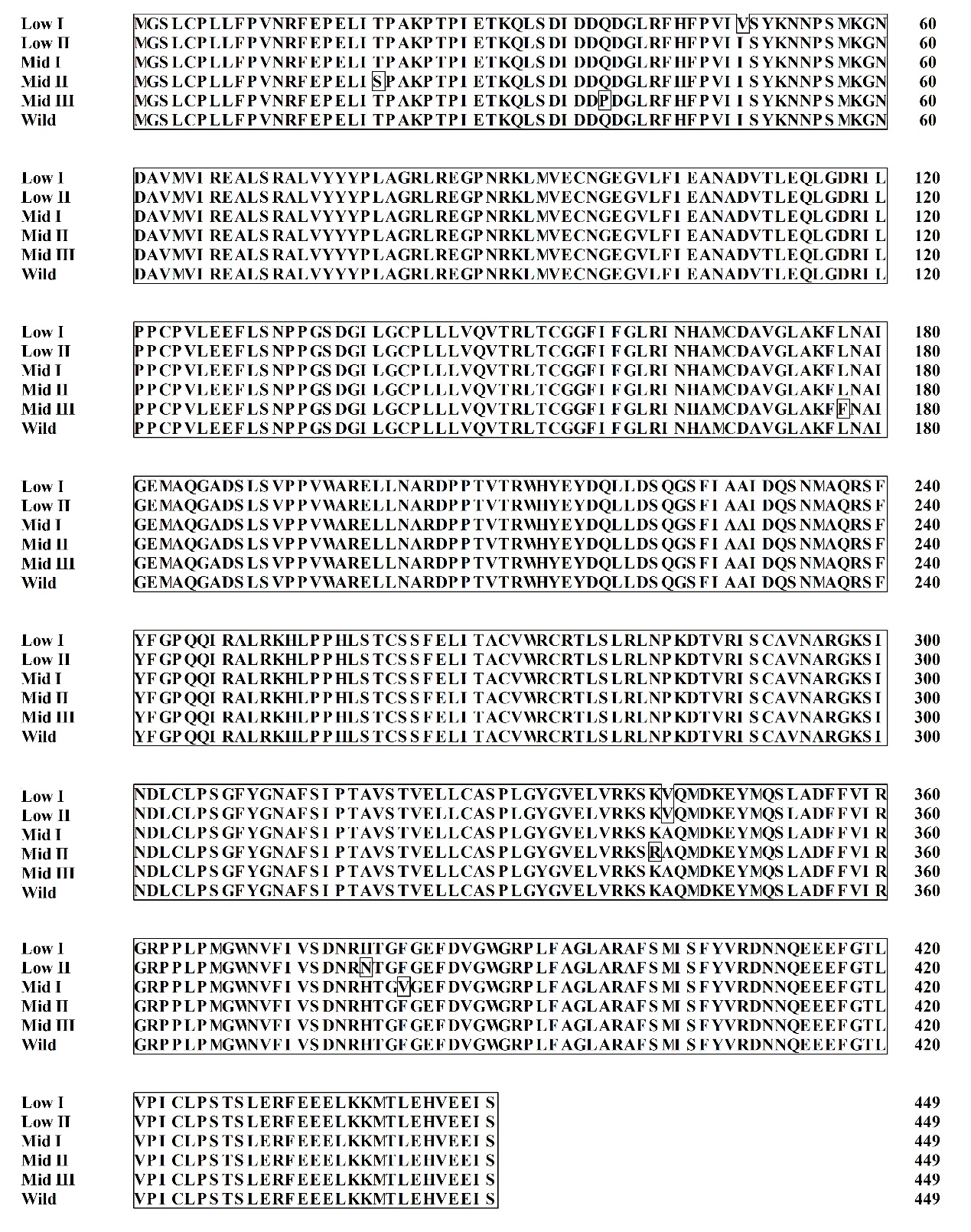


Fig. S1 Amino acid sequence differences of PpAAT1 among cultivars. The frames were the mutated amino acids. Low I: Low-aroma cultivar I; Low II: Low-aroma cultivar II; Low III: Low-aroma cultivar III; Mid I: Middle-aroma cultivar I; Middle II: Middle-aroma cultivar II; Middle III: Middle-aroma cultivar III; Wild: High-aroma cultivar.
